# Orbitrap Mass Spectrometry-Based Profiling of Secondary Metabolites in Two Unexplored *Eminium* Species and Bioactivity Potential

**DOI:** 10.3390/plants12122252

**Published:** 2023-06-08

**Authors:** Ebru Yuce Babacan, Dimitrina Zheleva-Dimitrova, Reneta Gevrenova, Abdelhakim Bouyahya, Mehmet Maruf Balos, Ugur Cakilcioglu, Kouadio Ibrahime Sinan, Gokhan Zengin

**Affiliations:** 1 Pertek Sakine Genç Vocational School, Munzur University, Pertek, Tunceli 62500, Turkey; ebruyuce@munzur.edu.tr (E.Y.B.); ucakilcioglu@yahoo.com (U.C.); 2 Department of Pharmacognosy, Faculty of Pharmacy, Medical University-Sofia, 1000 Sofia, Bulgaria; rgevrenova@pharmfac.mu-sofia.bg; 3 Laboratory of Human Pathologies Biology, Department of Biology, Faculty of Sciences, Mohammed V University in Rabat, Rabat 10106, Morocco; a.bouyahya@um5r.ac.ma; 4 Şanlıurfa Provincial Directorate of National Education, Karaköprü, Şanlıurfa 63320, Turkey; mbalos@gmail.com; 5 Physiology and Biochemistry Research Laboratory, Department of Biology, Science Faculty, Selcuk University, University Campus, Konya 42130, Turkey; sinankouadio@gmail.com (K.I.S.); gokhanzengin@selcuk.edu.tr (G.Z.)

**Keywords:** *Eminium*, cholinesterase, amylase, phenolics, phyto-pharmaceutics

## Abstract

The study aimed at the metabolite profiling and evaluation of antioxidant and enzyme inhibitory properties of methanol extracts from flowers, leaves, and tubers of unexplored *Eminium intortum* (Banks & Sol.) Kuntze and *E. spiculatum* (Blume) Schott (Araceae). A total of 83 metabolites, including 19 phenolic acids, 46 flavonoids, 11 amino, and 7 fatty acids were identified by UHPLC-HRMS in the studied extracts for the first time. *E. intortum* flower and leaf extracts had the highest total phenolic and flavonoid contents (50.82 ± 0.71 mg GAE/g and 65.08 ± 0.38 RE/g, respectively). Significant radical scavenging activity (32.20 ± 1.26 and 54.34 ± 0.53 mg TE/g for DPPH and ABTS) and reducing power (88.27 ± 1.49 and 33.13 ± 0.68 mg TE/g for CUPRAC and FRAP) were observed in leaf extracts. *E. intortum* flowers showed the maximum anticholinesterase activity (2.72 ± 0.03 mg GALAE/g). *E. spiculatum* leaves and tubers exhibited the highest inhibition towards α-glucosidase (0.99 ± 0.02 ACAE/g) and tirosinase (50.73 ± 2.29 mg KAE/g), respectively. A multivariate analysis revealed that *O*-hydroxycinnamoylglycosyl-*C*-flavonoid glycosides mostly accounted for the discrimination of both species. Thus, *E. intortum* and *E. spiculatum* can be considered as potential candidates for designing functional ingredients in the pharmaceutical and nutraceutical industries.

## 1. Introduction 

The search for bioactive natural compounds derived from medicinal plants is of great importance in modern medicine [1]. Natural products have been a rich source of pharmacologically active molecules that have been used to develop numerous drugs [2]. Furthermore, plant-derived compounds have been shown to have diverse biological activities, including antimicrobial, anti-inflammatory, and anticancer effects [3,4]. These compounds can serve as lead molecules for the development of new drugs or as alternative treatments to synthetic pharmaceuticals. Additionally, the use of natural products can help to reduce the potential side effects associated with synthetic drugs. In light of the growing interest in natural products, it is crucial to continue exploring the vast potential of medicinal plants to identify new and effective bioactive compounds that can benefit human health.

*Eminium* (Blume) Schott is a genus of the Araceae family, which includes over 300 species that grow mostly in tropical areas [5]. In Turkey, the Araceae family is represented by the main genera of *Arum, Biarum*, and *Eminium*. The genus *Eminium* includes seven taxa worldwide, five of which are distributed in Turkey, where its members are known locally as kardi, asalan, and kurtkulağı [6]. Studies on the biological activity and phytochemical constituents of *Eminium* are limited. Ethnobotanical records show that *Eminium* species are used for various purposes, including treating gastrointestinal problems [7], internal diseases, dysentery, and abdominal pain [8], yet there is a scarcity of phytochemical studies on the genus. In an earlier study by Afifi and Abu-Dahab [9], *E. spiculatum* (Blume) Schott was found to possess antimicrobial, antiplatelet, and antiproliferative properties, and the isolation of luteolin, luteolin-7-*O*-glucoside, chrysoeriol-7-*O*-glucoside, vitexin flowering, and isoorientin, as well as *β*-sitosterol from aerial parts of *E. spiculatum* was reported. Another study by Obeidat [10] reported antimicrobial properties of *E. spiculatum* against several strains of bacteria and fungi. Alkofahi et al. [11] reported the DNA protecting ability of the ethanolic fraction of *E. spiculatum*. To the best of our knowledge, there is no study on the chemical composition and bioactivity of *E. intortum* (Banks & Sol.) Kuntze.

The potential of the phytochemical screening strategy has been significantly increased by the recent progress of hyphenated techniques, which are able to afford efficient separation of the specialized metabolites and their dereplication at the same time. By coupling HPLC with mass spectrometry (LC/MS), especially high-resolution MS, a large amount of data can be obtained on the secondary metabolites of the plants’ extract before starting any isolation work [12].

Recently, ultra-high-performance liquid chromatography coupled with high resolution mass spectrometry (UHPLC-HRMS) has been introduced and become widespread in phytochemistry. HRMS is one of the most sensitive methods for analysis. Moreover, it has the potential to obtain information on the molecular weight, as well as on the structure of the secondary metabolites in plants. Due to its high potential of mass separation, very high selectivity can be obtained [12]. Thus, UHPLC-HRMS is a very suitable technique for analysis of unexplored plant species.

In light of the results of the literature review, we discovered a gap regarding the chemical profiles and biological activities of members of the genus *Eminium.* Therefore, we aimed to evaluate the biological properties of the methanolic extracts from three parts (leaves, flowers, and tubers) of two *Eminium* species (*E. intortum* and *E. spiculatum*). With regard to chemical profiles, the extracts were characterized by the UHPLC-HRMS (Orbitrap) technique. The results obtained could shed light on the biopharmaceutical potential of the member of the genus *Eminium*.

## 2. Results and Discussion

### 2.1. Total Phenol and Total Flavonoid Contents

The contents of total polyphenols (TP) and total flavonoids (TF) were measured for two plants, *E. intortum* and *E. spiculatum*, and the results are presented in Table 1. The three extracts from the different parts of both plants contained significant amounts of TP and TF, but the levels varied among the different plant parts and species studied. *E. intortum* was found to have higher levels of polyphenols and flavonoids, with the flower extract having the highest TP content (50.82 ± 0.71 mg GAE/g), followed by the leaves (25.86 ± 0.58 mg GAE/g) and tubers (21.31 ± 0.33 mg GAE/g). For flavonoids, *E. intortum* still had higher levels, with the leaf, flower, and tuber extracts containing 65.08 ± 0.38 mg GAE/g, 39.38 ± 0.65 mg GAE/g, and 9.81 ± 0.05 mg GAE/g, respectively.

Despite both plants belonging to the same genus and being harvested from the same places, *E. intortum* showed higher levels of TP and TF compared to *E. spiculatum*. This suggests that the differences in the chemical composition of these two plants are likely due to genetic factors.

Numerous studies have shown that the synthesis of polyphenols and flavonoids is regulated by genetics [13,14,15]. The flowers and leaves are also known to be the primary sites of storage and photosynthesis for these compounds [16,17,18]. This suggests that the high levels of TP and TF observed in *E. intortum* and *E. spiculatum* are likely due to their resistance to secondary metabolites, which is primarily achieved through the synthesis and storage of these compounds in leaves and flowers. In contrast, other parts of the plant, such as the tubers, typically have lower levels of polyphenols and flavonoids.

### 2.2. UHPLC-HRMS (Orbitrap) of Specialized Metabolites in Eminium Extracts

To estimate the specialized metabolites, non-targeted metabolic profiling of the carboxilic, phenolic, amino, and fatty acids, their derivatives, and flavonoids of both *E. intorum* and *E. spiculatum* extracts was carried out by UHPLC-Orbitrap-HRMS. Based on the MS and MS/MS accurate masses, fragmentation patterns, retention times, and comparison with reference standards and the literature data, a total of 83 metabolites, including 19 phenolic acids and derivatives, 46 flavonoids, 11 amino acids and derivatives, and 7 fatty acids were identified or tentatively annotated in *Eminium* extracts (Table 2).

#### Carboxylic, Hydroxybenzoic, Hydroxycinnamic, Acylquinic Acids, and Saccharides

Compound **1** [M-H]^−^ at *m*/*z* 341.109, corresponding to two ester bond hexoses gave fragment ions at *m/z* 179.055 [M-H-165.05]^−^, due to the loss of a hexosyl moiety. Fragment ions at *m*/*z* 119.033, 89.022, and 59.012 resulting from the sugar cross ring cleavages ^0,4^ Hex (−60 Da), ^0,3^ Hex (−90 Da), and ^0,2^ Hex (−120 Da) were registered, respectively. Thus, compound **1** was ascribed to sucrose [19]. Compound **2** [M-H]^−^ at *m*/*z* 191.018 showed fragment ions at 173.008 [M-H-H_2_O]^−^, 147.028 [M-H-CO_2_]^−^, a base peak at *m*/*z* 111.07 [M-H-CO_2_-2H_2_O]^−^, and was related to citric acid [20].

Three hydroxycinnamic acids (**12**, **13**, **18**, and **19**) together with chlorogenic acid (**9**) were identified in the *Eminium* extracts on the base of comparison with reference standards (Table 2). Six hydroxybenzoic glycosides were tentatively identified including hexosides (**3**, **4**, **5**, **16**, **17**) along with dihexoside of sinapic acid (**15**). In addition, hexosides of caffeic (**6**), coumaric (**11**), and ferulic acids (**14**), as well as their dihexosides (**7**, **8**, **10**) were annotated based on the diagnostic ions, corresponding to the loss of one (−162.05 Da) or two (−2 × 162.05 Da) hexoses (Table 2).

### 2.3. Flavonoids

Thirteen mono-, twenty-nine di-, and one triglycosides, as well as three aglycons were identified/annotated in the studied *Eminium* extracts (Table 2). Among them, forty were flavones, five flavonols, and one flavanone. The flavone aglycone apigenin (**63**), luteolin (**64**), and chrysoeriol (**65**) were deduced from the Retro-Diels-Alder (RDA) rearrangements ^1,3^ A^−^, ^0,4^ A^−^, ^1,2^A^−^, ^1,3^ B^−^, and ^1,2^ B^−^ (Table 2). Additionally, a series of neutral losses of CO_2_ (−44), CH_2_O (−30), CO (−28), and H_2_O (−18) supported the aglycone identification [21].

### 2.4. Flavonoid O-Glycosides

Peak dereplication of flavonoid *O*-glycosides was assigned by the neutral mass losses of 162.053, 308.112, and 324.106 Da consistent with hexose, rutinose/neohesperidose, and dihexose [22]. Thus, compounds **42**, **43**, **52**, **55**, **57**, and **58** were ascribed to *O*-hexosides of quercetin, luteolin, kaempferol, apigenin, chrysoeriol, and cirsiliol, respectively. Similarly, compounds **35**, **47**, **50**, and **56** were related to *O*-rutinosides of quercetin, kaempferol, isorhamnetin, and chrysoeriol. Compound **46** [M-H]^−^ at *m*/*z* 623.162 gave a base peak at *m*/*z* 299.056, corresponding to the concomitant loss of two hexoses, and was annotated as chrysoeriol 7-*O*-dihexoside. MS/MS spectrum of **54** [M-H]^−^ at *m*/*z* 577.1568 showed fragment ions at *m*/*z* 431.098 [M-H-dHex]^−^ and 413.088 [M-H-dHex-H_2_O]^−^, and a base peak at *m*/*z* 269.045 [M-H-dHex-Hex]^−^. The absence of the interglycosidic linkage breakdown is favored for 7-neohesperidoside. Thus, **54** was tentatively identified as apigenin 7-*O*-neohesperidoside (Table 2). Depending on the intensity and the ratio of the fragment ions [Y_0_]^−^ and [Y_0_-H]^−^, the sites for binding the sugar parts to the aglycones were also determined [23].

### 2.5. Flavonoid C-Glycosides

Key points in the dereplication of *C*-glycosides in the negative ion mode are fragment ions [(M-H)-120]^−^, [(M-H)-90]^−^, and [(M-H)-30]^−^. *C*-8 isomers were characterized by the base peak ^0,2^ X^−^ [(M-H)-120]^−^ as MS/MS of orientin (**30**), while *C*-6 isomers showed the base peaks [M-H]^−^ as homoorientin (**27**), isovitexin (**37**), and chrysoeriol 6-*C*-hexoside (**44**), together with abundant fragment ^0,2^ X^−^ [(M-H)-120]^−^ (about 60%). In addition, the fragment ion ^0,3^ X^−^ [M-H-90]^−^ was favored by *C*-6 isomers (Table 2) [24]. Compounds **20** and **22** gave indicative ions at *m*/*z* 519.113 and 503.119 [(M-H)-90]^−^, 489.103, and 473.109 [(M-H)-120]^−^, 429.082 and 413.089 [(M-H)-120-60]^−^, 399.072 and 383.077 [(M-H)-120-90]^−^, 369.061 and 353.066 [(M-H)-2 × 120]^−^, respectively. Based on ^1,3^ B^−^ fragments at *m*/*z* 133.028 (**20**) and 117.033 (**22**), **20** and **22** were tentatively annotated as 6,8-*C*-diglucosides of luteolin and apigenin, respectively (Table 2) [24].

### 2.6. Flavonoid C,O-Diglycosides

Among three isobars indicated as **20**, **21**, and **23** with [M-H]^−^ at 609.147, **21** and **23** were assigned to luteolin *C*, *O*-diglucosides. Typical ions of the *C*, *O*-flavon pathway resulted from the concomitant losses of hexosyl moiety (X_0_) and ^0,3^ X_1_ (−90) or ^0,2^ X_1_ (−120) at *m*/*z* 357.062 and 327.051, respectively (Table 2). In the same manner, **28** was ascribed as chrysoeriol *C*, *O*-diglucoside. Chrysoeriol was deduced from the prominent fragment ions at *m*/*z* 327.051 Y_0_^−^/^0,2^ X_1_/CH_2_, 299.0542 [chrysoeriol-H]^−^, 298.048 Y_0_^−^/^0,2^ X_1_/CH_2_/CHO, 297.041 Y_0_^−^/^0,2^ X_1_/·CH_3_/CO, and 269.046 Y_0_/^0,2^ X_1_/·CH_3_/2CO. Apigenin *O*-pentosyl-6-*C*-hexoside (**33**) was discernable by the fragment ions at *m/z* 341.067 ([(M-H)-132-90]^−^ and 311.0561 ([(M-H)-132-120]^−^, suggesting the presence of both *O*-pentosyl (X_1_) and *C*-hexosyl (X_0_) moieties. Additionally, the aforementioned structure was assigned on the basis of a series of fragment ions at 283.061 (Y_1_^−^/^0,2^ X_0_/CO)_,_ 282.055 (Y_1_^−^/^0,2^ X_0_/CHO), 281.045 (Y_1_^−^/^0,2^ X_0_/CH_2_O), and 237.055 (Y_1_^−^/^0,2^ X_0_/2CO) (Table 2).

**Figure 1:**
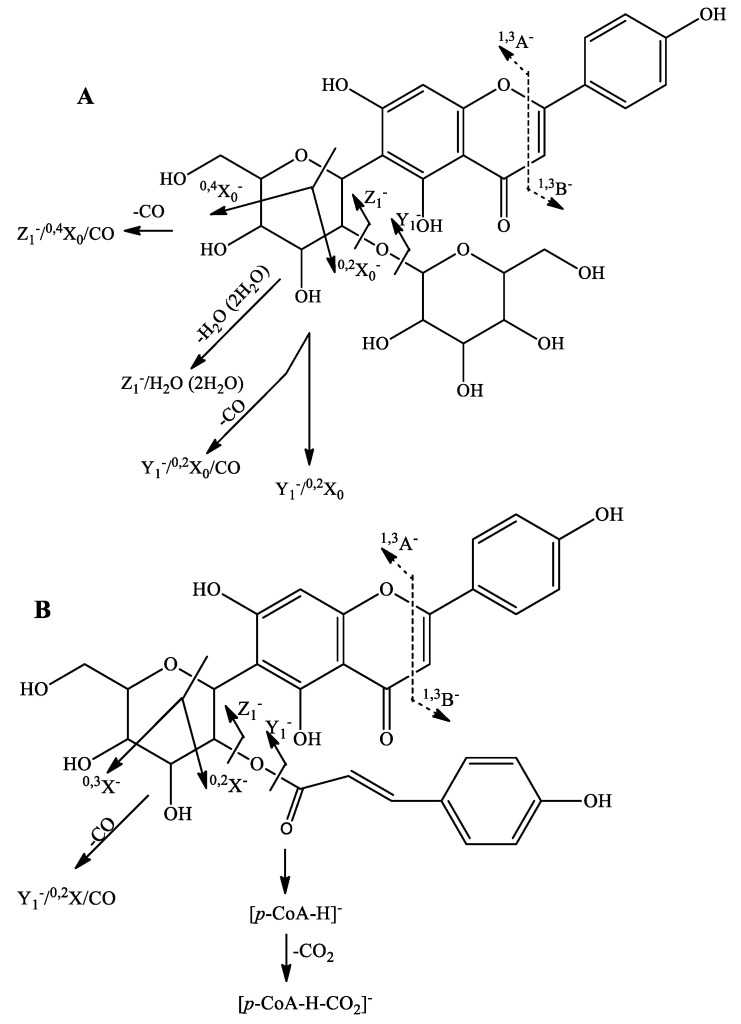
Possible fragmentation pathway of (**A**) apigenin 2″-*O*-hexosyl-6-*C*-hexoside (**32**) and (**B**) apigenin 2″-*O*-coumaroyl-6-*C*-hexoside (**61**).

MS/MS spectrum of **29** with ([M-H]^−^ at *m*/*z* 771.179 was acquired. In (-) ESI mode, **29** yielded prominent fragment ions at *m*/*z* 447.093 [(M-H)-2 × 162.05]^−^, 357.062 [(M-H)-2 × 162.05-90]^−^, 327.051 ([(M-H)-2 × 162.05-120]^−^, suggesting the presence of both *O*-dihexosyl and *C*-hexosyl moieties. The absence of the interglycosidic linkage breakdown is favored for *O*-sophoroside. Thus, **29** was annotated as luteolin *O*-sophoroside-6-*C*-hexosyl-orientin. Compound **32** ([M-H]^−^ at *m*/*z* 593.151) yielded prominent ions at *m*/*z* 413.088 ([(M-H)-(162.05 + H_2_O)]^−^, 353.067 ([(M-H)-(162.05 + H_2_O)-60]^−^, 311.056 ([(M-H)-(162.05)-120]^−^, 293.047 [(M-H)-(162.05 + H_2_O)-120]^−^, and 281.045 Y_1_^−^/^0,2^ X_0_/CH_2_O indicating an *O*-hexosyl unit at the 2”of the primary hexose [24]. Thus, **32** was assigned to 2″-*O*-hexosyl-6-*C*-hexosyl-apigenin (Figure 1).

**Figure 2:**
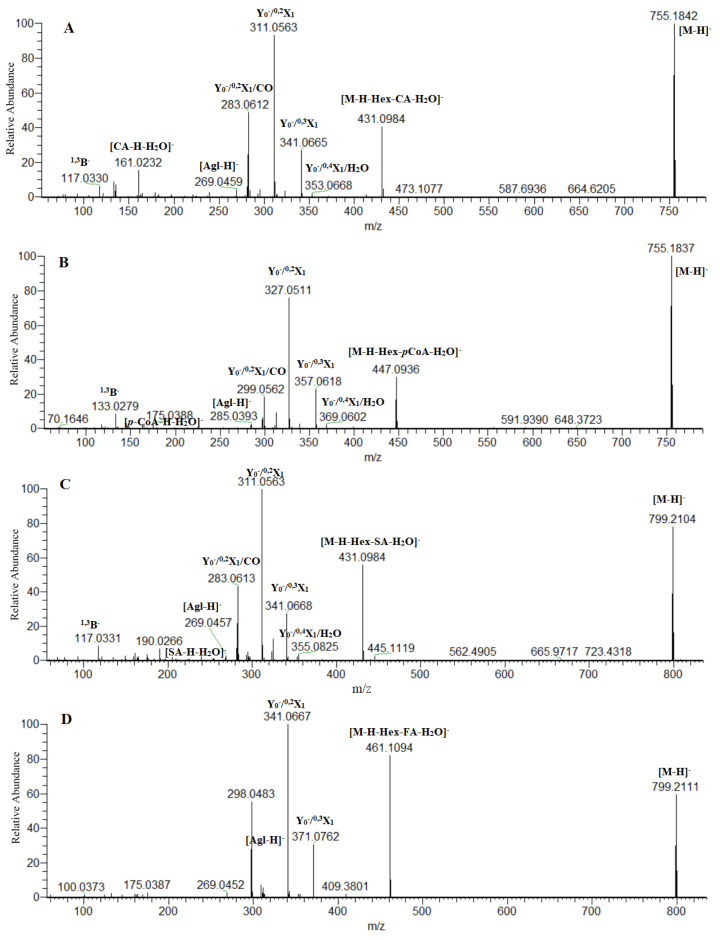
(-) ESI/MS-MS spectrum of apigenin *O*-caffeoylhexosyl-8-*C*-hexoside (**36**) (**A**), luteolin 7-*O*-coumaroylhexosyl-6-*C*-hexoside (**39**) (**B**), apigenin 7-*O*-synapoylhexosyl-8-*C*-hexoside (**45**) (**C**), chrysoeriol *O*-feruloylhexoside-8-*C*-hexoside (**49**) (**D**); CA-caffeic acid; *p*-CoA-*p*-coumaric acid; SA-sinapic acid; FA-ferulic acid.

Two compounds **34** ([M-H]^−^ at *m*/*z* 815.205) and **45** ([M-H]^−^ at *m*/*z* 799.210) shared the same fragmentation patterns yielding prominent ions at *m*/*z* 447.094 (**34**) and 431.098 (**35**), respectively, indicating a concomitant loss of hexose (162.05 Da) and sinapoyl residue (206 Da, C_11_H_10_O_4_) (Table 2). Moreover, sinapoyl moiety was evidenced by the fragment ions at *m*/*z* 205.049 [(sinapic acid-H)-H_2_O]^−^, 190.027 [(sinapic acid-H)-H_2_O-CH_3_]^−^, and 175.003 [(sinapic acid-H)-H_2_O-2·CH_3_]^−^. The presence of a *C*-hexosyl moiety on the flavonoid skeleton was deduced from the relative abundances of the ions at *m*/*z* 327.051 [M-H-120]^−^ (79.7%) (**34**) and 311.056 (100%) (**45**) suggesting a 6-*C*- and an 8-*C*-linkage, respectively. Thus, **34** was ascribed as luteolin *O*-sinapoylhexosyl-6-*C*-hexoside, while **45** was annotated as apigenin *O*-sinapoylhexosyl-6-*C*-hexoside (Figure 2).

Two caffeoyl esters **36** and **41** were discernable by the transition [M-H]^−^→Y_0_ resulting from the indicative loss of 324.085 Da (C_15_H_16_O_8_). Precursor ions gave a series of fragment ions involved in the *C*-glycosyl flavon pattern: *m*/*z* 353.064 (Y_0_^−^/^0,4^ X_1_/H_2_O), 341.067 (Y_0_^−^/^0,3^ X_1_), 311.056 (Y_0_^−^/^0,2^ X_1_), and 283.061 (Y_0_^−^/^0,2^ X_1_/CO) (Table 2). Caffeoyl residue was evidenced by the diagnostic ions at *m*/*z* 161.023 [(caffeic acid-H)-H_2_O]^−^ and 133.028[(caffeic acid-H)-H_2_O-CO]^−^. Accordingly, **36** and **41** were assigned to apigenin *O*-caffeoylhexosyl-8-*C*-hexoside and chrysoeriol *O*-caffeoylhexosyl-8-*C*-hexoside (Figure 2).

Feruloyl esters of luteolin-, apigenin-, and chrysoeriol *O*-hexosyl-8-*C*-hexoside (**38**, **48**, and **49**) were deduced from the loss of feruloylhexosyl residue (338 Da, C_16_H_18_O_8_) and the subsequent transition of *m*/*z* 447.094/431.099/461.109→327.051/311.056/341.067, respectively, arising from the hexose cross ring cleavage (^0,2^ X_1_) (Table 2). The ions at *m*/*z* 175.039 [(ferulic acid-H)-H_2_O]^−^, 160.015 [(ferulic acid-H)-CH_3_]^−^, and [(ferulic acid-H)-H_2_O-CH_3_-CO]^−^ point out to the feruloyl moiety (Table 2) (Figure 2).

In the same way, **39**, **48**, and **53** were ascribed as coumaroyl esters of luteolin-, apigenin-, and chrysoeriol *O*-hexosyl-*C*-hexoside. The commonly found loss of 308 Da (C_15_H_16_O_7_) in the fragmentation patterns of the aforementioned compounds, accompanied with the ions at *m*/*z* 163.039 [*p*-coumaric acid-H]^−^, 145.028 [(*p*-coumaric acid-H)-H_2_O]^−^, and 117.033 [(*p*-coumaric acid-H)-H_2_O-CO]^−^ confirmed the presence of a coumaroyl moiety (Figure 2). Two coumaroyl esters of luteolin- and apigenin-*O*-deoxyhexosyl-6-*C*-hexoside (**60** and **62**) were evidenced on the base of the loss of 292 Da (C_15_H_16_O_6_) (Table 2) (Figure 1).

The identification of flavonoids **25**, **27**, **30**, **35**, **37**, **42**, **47**, **50**, **52**, **55**, **63**, and **64** was confirmed by comparison with reference standards.

### 2.7. Amino Acids and Derivatives

Based on the comparison with the literature data, two amino acids (**71** and **73**), a dipeptide (**74**), and six amino acid hexosides were tentatively annotated (Table 2). Compound **71** [M-H]^−^ at *m*/*z* 164.071 gave a base peak at *m*/*z* 147.043 [M-H-NH_3_]^−^, and prominent ions at *m*/*z* 146.059 [M-H-H_2_O]^−^, 119.048 [M-H-NH_3_-CO]^−^, and 103.918 [M-H-NH_3_-CO-CH_3_]^−^. Thus, **71** was assigned as phenylalanine [19]. Compound **73** with the molecular formula C_11_H_12_O_2_N_2_ showed fragments at *m*/*z* 186.054 [M-H-NH_3_]^−^, 159.091 [M-H-NH_3_-CO_2_]^−^, 142.064 [M-H-2NH_3_-CO_2_]^−^, and a base peak at *m*/*z* 116.049 [M-H-2NH_3_-CO_2_-C_2_H_2_]^−^. Thus, **73** was annotated as tryptophan; an essential amino acid previously described in the Araceae family [19,25]. MS/MS spectrum of **74** (C_11_H_20_O_5_N_2_), a series of fragment ions resulting from neutral losses were registered at *m*/*z* 241.119 [M-H-H_2_O]^−^, 223.108 [M-H-2H_2_O]^−^, 215.140 [M-H-CO_2_]^−^, 197.1287 [M-H-H_2_O-CO_2_]^−^, and a base peak at *m*/*z* 128.033, corresponding to the loss of leucine [M-H-131.096]^−^ from the glutamyl residue. Thus, **74** was related to γ-glutamyl-leucine [19]. MS/MS spectra of compounds **66**, **67**, **68**, **69**, **70**, and **72** demonstrated base peaks, resulting from the loss of a hexose moiety, with the appearance of the corresponding amino acid residue. Hence, they were annotated as hexosides of glutamic acid, valine, tyrosine, leucine, phenylalanine, and tryptophan, respectively (Table 2) [19].

### 2.8. Fatty Acids

Based on the comparison of accurate masses and MS/MS fragmentation patterns with the literature data, one saturated (**82**), three monounsaturated (**76**, **79**, and **80**), and five polyunsaturated (**75**, **77**, **78**, **81**, and **83**) free fatty acids were tentatively annotated in *Eminium* extracts (Table 2) [25]. Among them, five (**78**–**82**) were dihydroxylated and three (**75**–**77**) contained three hydroxyl groups (Table 2). Compound **83** was dereplicated as linoleic acid, previously found in *E. rauwolffii* [26].

## 3. Antioxidant Activity

To study the antioxidant activity of *E. intortum* and *E. spiculatum* methanolic extracts, we used six different methods including DPPH, ABTS cation, FRAP, CUPRAC, phosphomolybdenum, and metal chelate assays. As shown in Table 3, all the extracts tested showed significant radical scavenging activity. The methanolic extracts of *E. intortum* exhibited a much stronger antioxidant activity compared to the extracts of *E. spiculatum*, with values of 32.20 ± 1.26 mg TE/g for leaves, 27.87 ± 0.75 mg TE/g for flowers, and 26.90 ± 1.61 mg TE/g for tubers. The methanolic extract of the tuber of *E. spiculatum* also showed good antioxidant activity with a value of 33.67 ± 1.26 mg TE/g.

Table 3 illustrates the ability of the extracts to scavenge the ABTS cation. The methanolic fractions of all studied parts of both plants had similar significant scavenging activity against ABTS. According to the CUPRAC method, the methanolic extract of the leaves of both plants showed significant antioxidant activity, with a value of 88.27 ± 1.49 for *E. intortum* and 86.27 ± 2.74 for *E. spiculatum*. The methanolic extracts of the tubers and flowers of both plants showed lower antioxidant activity compared to the leaves.

In the FRAP assay, a high absorbance indicates a high reducing power. The methanolic extracts of the tubers of both *E. spiculatum* and *E. intortum* showed very significant reducing activity, with values of 41.33 ± 1.25 mg TE/g and 32.58 ± 4.02 mg TE/g, respectively. The other extracts of both plants also showed significant results of antioxidant activity in this assay. In summary, our findings suggest that the extracts are rich in phenolic compounds with radical-scavenging activity and proton-donating ability. Polyphenols have been acknowledged for their antioxidant activity, which may account for their potential ability to prevent various diseases associated with oxidative stress. This assertion is supported by several studies [27,28,29,30]. When using the phosphomolybdenum method, all plant extracts displayed low levels of antioxidant activity with comparable values, as this method is specific in nature. Conversely, when using the metal chelation method, significant effects were observed in the methanolic extracts of the leaves of both plants, with values of 63.43 ± 0.70 mg EDTAE/g for *E. intortum* and 61.55 ± 3.97 mg EDTAE/g for *E. spiculatum*. It should be noted that this method is also highly specific.

The antioxidant activity of a substance is influenced by how it interacts with radicals in the reaction medium. These interactions are facilitated by active molecules that trap the radicals, and previous studies have highlighted their importance [31,32,33]. The efficacy of antioxidants is not solely dependent on the concentration of the main constituents but also on the presence of other constituents in smaller amounts, or the synergy between the constituents [34,35,36,37].

Limited literature exists regarding the antioxidant properties of the *Eminium* genus. Alkofahi et al. [11] conducted a study demonstrating the protective effect of the *E. spiculatum* extract against oxidative DNA damage using the 8-hydroxydeoxyguanosine assay, while no beneficial effect on alleviating oxidative damage was observed. Al-Ismail et al. [38] reported the antioxidant properties of the *E. spiculatum* ethanolic extract using DPPH, FRAP, and vegetable oil emulsion systems, where the extract exhibited significant properties with lower IC_50_ values. Additionally, Janat and Al-Thnaibat [39] reported that the methanolic extract of *E. spiculatum* exhibited higher antioxidant activity in the phosphomolybdenum assay when compared to the aqueous extract.

## 4. Enzymes Inhibitory Activities

### 4.1. α-Amylase and α-Glucosidase Inhibition

In the intestinal tract, the breakdown of complex sugars into simple sugars is facilitated by two essential enzymes, namely α-amylase and α-glucosidase. The simple products, particularly glucose, are subsequently absorbed and can cause a rise in blood sugar levels. To manage diabetes, one potential therapeutic approach is to inhibit the enzymes responsible for carbohydrate hydrolysis, which can decrease postprandial blood sugar levels [40,41]. By inhibiting these enzymes in the intestinal tract, the degradation of complex sugars into simple sugars can be prevented, leading to a reduction in blood sugar levels [42,43,44]. In our study, we investigated the potential inhibition of these enzymes by extracts from the two plants.

Table 4 shows the inhibitory effects of methanolic extracts from various parts of the two plants on α-amylase and α-glucosidase. Our results indicate that these extracts have the potential to inhibit the activity of both enzymes. Interestingly, the methanolic extracts of leaves and flowers from *E. spiculatum* also exhibited α-glucosidase inhibition, with values of 0.99 ± 0.02 ACAE/g and 0.89 ± 0.01 ACAE/g, respectively. Among *E. intortum* extracts, the leaf extract was only active against α-glucosidase (0.35 mmol ACAE/g). In addition, both tuber extracts showed no inhibitory effect on α-glucosidase. Our results demonstrate the potential of the studied leaf extracts to act as inhibitors of carbohydrate hydrolyzing enzymes. By delaying the absorption of dietary carbohydrates in the small intestine and reducing postprandial hyperglycemia, these inhibitors may be a valuable component in the development of antidiabetic drugs. This mechanism of action has been previously reported [45,46,47].

Our study is the first to investigate the antidiabetic activity of the two plants, and we found that all studied extracts of the plants inhibited α-amylase activity, with values lower than 0.31 ± 0.01 ACAE/g. The inhibitory effect of phenolic compounds on carbohydrate hydrolysis enzymes has been reported previously [48,49,50], and this may be due to their ability to bind to proteins.

#### 4.1.1. Cholinesterase Inhibition

Acetylcholinesterase (AChE) and butyrylcholinesterase (BChE) are enzymes found primarily in nerve tissues and neuromuscular junctions. They are responsible for rapidly hydrolyzing acetylcholine, a neurotransmitter, into inert choline and acetate. Overexpression or excessive catalysis of AChE can cause neuronal disturbances and lead to neurological disorders. A potential strategy for neuroprotection is to inhibit AChE activity [51,52,53]. Our research aimed to investigate the potential inhibitory effect of our plant extracts on the enzymes AChE and BChE. The results of this study are presented in Table 3, and they show that the extracts were able to inhibit both AChE and BChE. Specifically, the methanolic extract of both plants exhibited significant inhibitory activity against AChE, with values ranging from 1.27 ± 0.09 mg GALAE/g to 2.72 ± 0.03 mg GALAE/g. Interestingly, most of the extracts displayed greater inhibitory power on AChE than on BChE. It is worth noting that cholinesterase inhibitors are known for their therapeutic action in inhibiting acetylcholinesterase at the central level, and our findings suggest that our plant extracts could potentially be used for this purpose. Numerous studies have reported that various plant species possess the ability to block and inhibit both types of enzymes (AChE and BChE) [54,55,56].

#### 4.1.2. Tyrosinase Inhibition

In skin cells, tyrosinase plays a crucial role in the aging process, and inhibiting its activity is a key strategy for delaying skin aging. This enzyme catalyzes the initial two steps of melanogenesis, making it a rate-limiting factor in this process. Mutations in the tyrosinase gene or its absence can cause a reduction or cessation of pigmentation [57,58]. In our study, we tested all the extracts against tyrosinase and demonstrated that they all exhibit activity, with variation depending on the part studied and the solvent used. The results are presented in Table 3. The methanolic extracts of all three parts of each plant showed very strong tyrosinase inhibitory activity. However, the methanolic extract of *E. spiculatum and E. intortum* tubers exhibited strong inhibitory activity with values of 50.73 ± 2.29 KAE/g and 48.13 ± 0.24 KAE/g, respectively. Natural compounds capable of inhibiting tyrosinase activity are of great interest, with increasing demand in the fields of cosmetics and pharmaceuticals [59,60]. Numerous studies have revealed the tyrosinase inhibitory activity of several plant species, which have demonstrated a significant effect against this enzyme [61,62,63,64,65].

#### 4.1.3. Data Analysis

**Figure 3:**
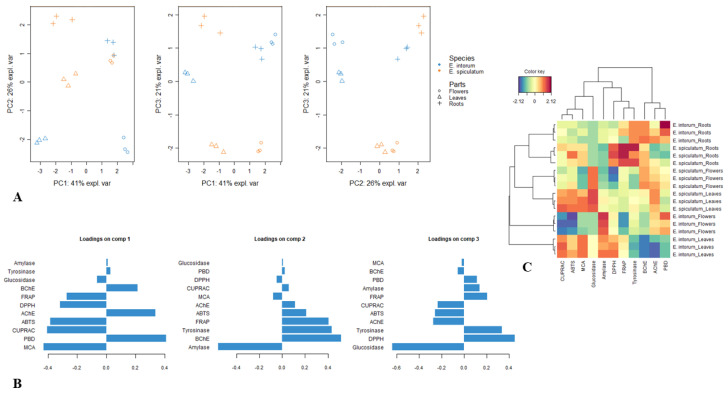
Exploratory Principal Component Analysis. (**A**). Scatter plot showing the distribution of the samples in the factorial plan derived from the three retained dimensions. (**B**). Loading plots showing the relationship of biological activities on each dimension of PCA. (**C**). Clustered Image Map on biological activities dataset. (Blue color: low bioactivity. Red color: High bioactivity).

To gain more insight into the tested extracts, we evaluated the results of chemical components and bioactivity assays using multivariate analysis. In recent years, multivariate analysis has become increasingly popular in phytochemical studies as it helps establish connections between different parameters. By determining a small number of principal components based on Kaiser’s rule, we performed PCA analysis using three components. The first component (PC1) was mainly associated with antioxidant assays and AChE. The second component (PC2) contained amylase, BChE, and tyrosinase, while the third component (PC3) included glucosidase and DPPH (Figure 3B). Although the distribution of the samples on the score plot generated from the three principal components exhibited variability, it was challenging to clearly distinguish between different homogenous groups (Figure 3A). A multivariate analysis has provided more information about the connections between chemical profiles and biological properties, as documented in the literature. To classify the samples and identify the biological activities that characterize each cluster, we created a heat map. Based on the heat map, we obtained four clusters. The tuber samples were classified in the same cluster (Cluster I), while Cluster II contained the flowers and leaves of *E. spiculatum*. In contrast, the flower and leaves of *E. intortum* were grouped into Cluster III and IV, respectively (Figure 3C).

**Figure 4:**
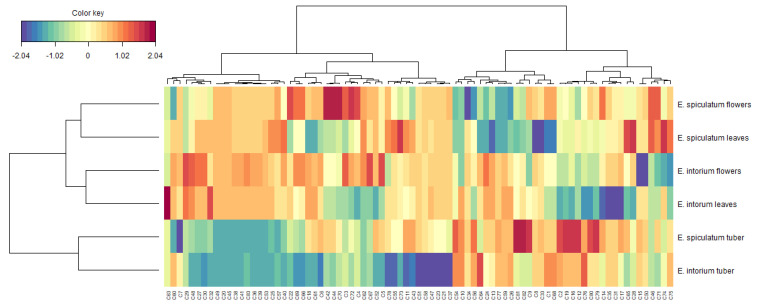
Global overview of the secondary metabolites’ contrasts among *Eminium* samples (Blue color: low content. Red color: high content). For compound numbers refer to Table 1.

**Figure 5:**
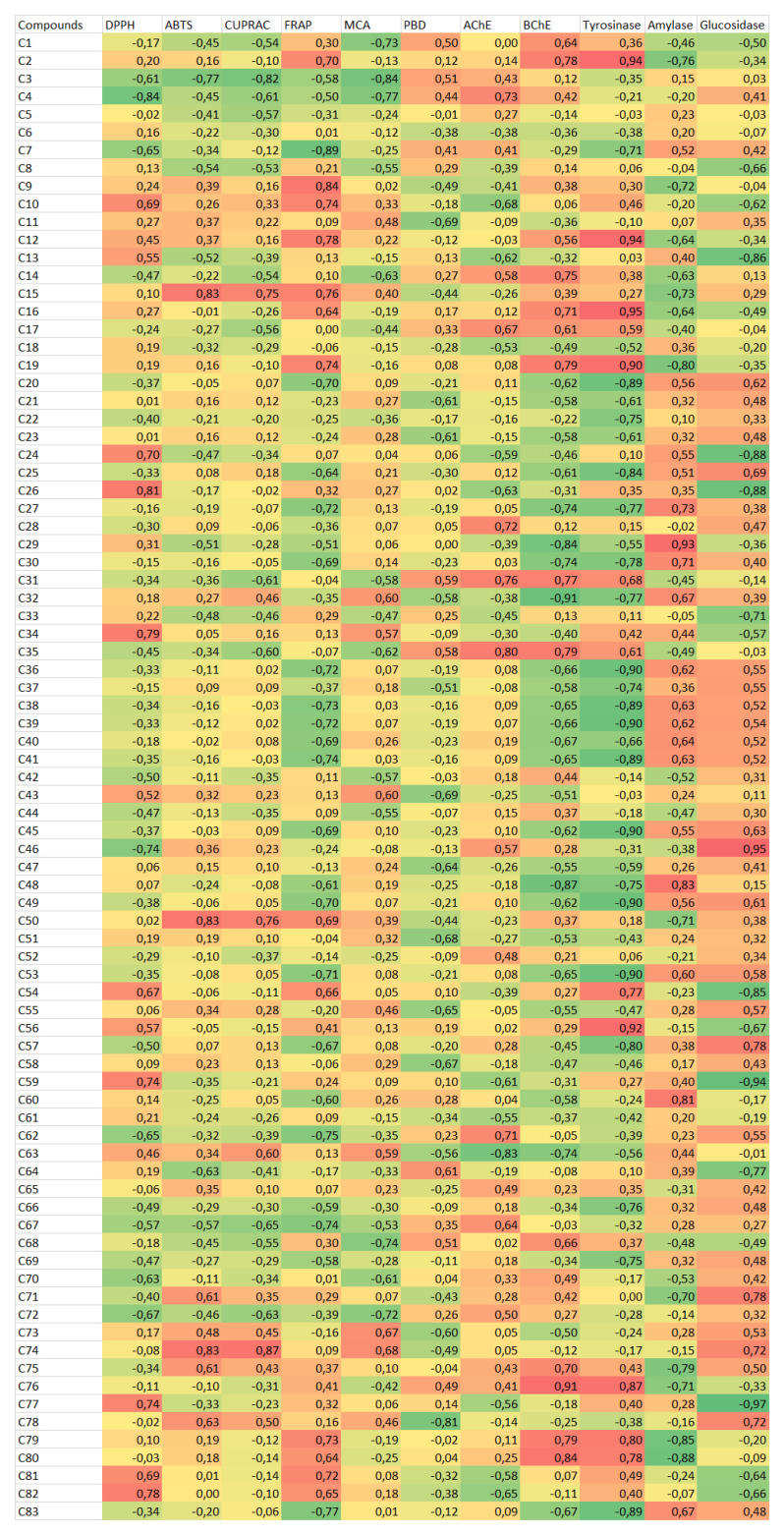
Relationship between polyphenol metabolites and antioxidant activities of samples. For compouns numbers refer to Table 1.

The classifications of the tested extracts based on their chemical components is presented in Figure 4. Cluster I included the leaves and flowers of *E. spiculatum*, which contained high levels of chrysoeriol *O*-coumaroylhexoside-8-*C*-hexoside, luteolin-6,8-*C*-diglucoside, and apigenin 7-*O*-synapoylhexosyl-8-*C*-hexoside. The leaves and flowers of *E. intortum* were classified in Cluster II, which were characterized by high levels of luteolin 7-*O*-coumaroylhexosyl-6-C-hexoside and apigenin *O*-feruloylhexosyl-8-*C*-hexoside. Cluster III contained both tuber samples. Furthermore, we conducted a correlation analysis between the biological activities and individual components, and the results are shown in Figure 5. Some compounds were found to be strongly correlated with the tested biological activities. For instance, apigenin-6-*C*-hexoside-8-*C*-pentoside was the main contributor to DPPH scavenging ability, while sinapic acid-*O*-dihexoside and isorhamnetin-3-*O*-rutinoside were positively correlated with ABTS and CUPRAC. Consistent with our findings, the compounds have been described as important antioxidants by several researchers [66,67,68]. Additionally, rutin was found to be the primary player in AChE and BChE assays, consistent with previous studies [69].

## 5. Materials and Methods

### 5.1. Plant Materials

In the summer of 2021, we collected *Eminium* species in Turkey (*Eminium intortum* (Banks & Sol.) Kuntze: Between Kızıltepe and Mardin, 533 m, GPS: 37°14′20″ N, 40°38′10″ E; *Eminium spiculatum* (Blume) Schott: Derik, Mardin, 661 m, GPS: 37°18′44″ N, 40°16′16″ E). The plant specimens were identified by one of our co-authors, Dr. Ugur Cakilcioglu, and one specimen from the plants was deposited at the Harran University herbarium. Prior to extraction, the plant materials were carefully washed with tap and distilled water to eliminate any soil and contaminants. After being air-dried for 10 days (in shade at room temperature), the flowers, leaves, and roots were powdered.

### 5.2. Extraction of Samples

For extraction, we employed the maceration method, in which 5 g of plant material was mixed with 100 mL of methanol and left to macerate at room temperature for 24 h. After maceration, the extracts were filtered through Whatman filter paper, and the solvents were evaporated using a rotary-evaporator. To preserve the extracts, we stored them at 4 °C until analysis.

### 5.3. Chemicals

Acetonitrile (hypergrade for LC–MS), formic acid (for LC–MS) and methanol (analytical grade) were purchased from Merck (Merck, Bulgaria). The reference standards used for compound identification were obtained from Extrasynthese (Genay, France) for *p*-coumaric, *o*-coumaric, ferulic acids, saponarin, homoorientin, orientin, rutin, isovitexin, isoquercitrin, luteolin 7-*O*-glucoside, kaempferol 3-*O*-rutinoside, kaempferol 3-*O*-glucoside, isorhamnetin 3-*O*-glucoside, apigenin 7-*O*-glucoside, quercetin, and apigenin. Chlorogenic and caffeic acids were supplied from Phytolab (Vestenbergsgreuth, Bavaria, Germany).

### 5.4. Total Quantification of Phenolics and Flavonoids

We determined the total phenolic and flavonoid content of the extracts using the Folin–Ciocalteu and AlCl3 assays, respectively, according to Zengin and Aktumsek’s protocol (2014). The results of these tests were reported in terms of gallic acid equivalents (mg GAE/g dry extract) and rutin equivalents (mg RE/g dry extract) [70].

### 5.5. UHPLC-HRMS (Orbitrap)

The UHPLC-HRMS analyses were carried out on a Q Exactive Plus mass spectrometer (ThermoFisher Scientific, Inc.) equipped with a heated electrospray ionization (HESI-II) probe (ThermoScientific). The equipment was operated in negative and positive ion modes within the *m*/*z* range from 100 to 1000. The mass spectrometer parameters were as follows: spray voltage 3.5 kV (+) and 2.5 kV (−); sheath gas flow rate 38; auxiliary gas flow rate 12; spare gas flow rate 0; capillary temperature 320 °C; probe heater temperature 320 °C; S-lens RF level 50; scan mode: full MS (resolution 70,000), and MS/MS (17,500). The chromatographic separation was achieved on a reversed phase column Kromasil EternityXT C18 (1.8 µm, 2.1 × 100 mm) at 40 °C. The UHPLC analyses were run with a mobile phase consisting of 0.1% formic acid in water (A) and 0.1% formic acid in acetonitrile (B). The run time was 33 min. The flow rate was 0.3 mL/min. The gradient elution program was used as follows: 0–1 min, 0–5% B; 1–20 min, 5–30% B; 20–25 min, 30–50% B; 25–30 min, 50–70% B; 30–33 min, 70–95%; 33–34 min 95–5% B. Equilibration time was 4 min [22]. Data were processed by Xcalibur 4.2 (ThermoScientific, Waltham, MA, USA) instrument control/data handling software. Metabolite profiling using MZmine 2 software was applied to the UHPLC–HRMS raw files of the studied extracts. The areas under the curve (AUC) for each identified compound were plotted and used for further statistical analysis in 3.10 [22].

### 5.6. Assays for Antioxidant and Enzyme Inhibition

We analyzed the extracts for a range of antioxidant and enzyme inhibitory activities, including, cupric reducing antioxidant capacity (CUPRAC), DPPH, and ABTS radical scavenging, metal chelating activity (MCA), ferric reducing antioxidant power (FRAP), phosphomolybdenum (PBD), and inhibition of amylase, tyrosinase, glucosidase, acetylcholinesterase (AChE), and butyrylcholinesterase (BChE). We employed the previously described methods to evaluate these activities [71]. Each sample was analyzed three times.

### 5.7. Data Analysis

All data were given as mean ± standard deviation (SD). Statistical analysis was performed by analysis of variance (ANOVA). A post hoc test (Tukey) was performed when the differences shown by data were significant (*p* < 0.05). Then, Principal Component Analysis (PCA) and hierarchical clustered analysis (HCA) were performed to emphasize the distinct clusters in terms of their bioactivities. Furthermore, hierarchical clustered analysis (HCA) was performed to assess the (dis)similarity between samples in terms of their molecules. All used data were scale and molecules data and were log transformed before doing multivariate analysis. R v.4.2.3 statistical program was used for all analyses.

## 6. Conclusions

In our study, we explored the detailed chemical composition and biological effects of two *Eminium* species: *E. intortum* and *E. spiculatum*. We found that the chemical composition and biological properties (antioxidant and enzyme inhibitory effects) varied according to the plant parts used. Generally, the leaf extract of both species exhibited higher antioxidant effects compared to flowers and tubers. However, we obtained different results for each enzyme inhibition assay, and the leaf extracts provided good anti-amylase and anti-glucosidase actions. The extracts were rich in flavonoids. These findings provide a valuable scientific basis for evaluating the potential of the *Eminium* genus and suggest that the tested species could be considered as a source of natural bioactive agents for health-promoting applications. Nevertheless, additional research is necessary to elucidate the potential toxicity of both the extracts and the individual chemical constituents.

## Figures and Tables

**Table 1:** Total phenolic (TP) and flavonoid (TF) content of the tested extracts.

Species	Parts	TP (mg GAE/g)	TF (mg RE/g)
*Eminium intortum*	Leaves	25.86 ± 0.58 ^b^	65.08 ± 0.38 ^a^
	Flowers	50.82 ± 0.71 ^a^	39.38 ± 0.65 ^b^
	Tubers	21.31 ± 0.33 ^c^	9.81 ± 0.05 ^c^
*Eminium spiculatum*	Leaves	23.90 ± 0.57 ^a^	46.54 ± 0.40 ^a^
	Flowers	21.30 ± 1.25 ^b^	19.91 ± 0.15 ^b^
	Tubers	18.68 ± 0.34 ^c^	3.93 ± 0.12 ^c^

Values are reported as mean ± SD of three parallel measurements. GAE: Gallic acid equivalent; RE: Rutin equivalent. Different letters indicate significant differences between the tested extracts (*p* < 0.05).

**Table 2:** Secondary metabolites in *Eminium* extracts.

№	Identified/Tentatively Annotated Compound	Molecular Formula	Exact Mass [M-H]^−^	Fragmentation Pattern in (-) ESI-MS/MS	t_R_ (min)	Δ ppm	Distribution
Carboxylic, hydroxybenzoic, hydroxycinnamic, acylquinic acids, and saccharides							
**1.**	sucrose	C_12_H_22_O_11_	341.1089	341.1089 (100), 179.0550 (46.7), 161.0444 (12.4), 143.0337 (10.1), 131.0336 (5.7), 119.0335 (36.7), 101.0228 (39.3), 89.0228 (91.1), 71.0122 (75.6), 59.0122 (57.5)	0.68	−0.043	1,2,3,4,5,6
**2.**	citric acid	C_6_H_8_O_7_	191.0189	191.0189 (9.6), 173.0080 (1.5), 147.0287 (0.5), 129.0179 (5.9), 111.0072 (100), 87.0071 (47.8),	0.91	−4.428	1,2,3,4,5,6
**3.**	salicylic acid *O*-hexoside	C_13_H_16_O_8_	299.0779	137.0230 (100), 93.0330 (63.1)	1.28	2.037	1,2,3,4,5,6
**4.**	protocatechuic acid-*O*-hexoside	C_13_H_16_O_9_	315.0723	315.0723 (100), 153.0181 (25.7), 152.0103 (56.8), 109.0289 (7.3), 108.0201 (81.9), 123.0071 (3.9)	1.64	0.459	1,2,3,4,5,6
**5.**	salicylic acid *O*-hexoside isomer	C_13_H_16_O_8_	299.0772	299.0772 (100), 239.0557 (20.5), 209.0449 (3.1), 179.0339 (40.4), 137.0230 (97.6)	2.04	−0.102	1,2,3,4,5,6
**6.**	caffeic acid*-O* hexoside O-caffeoylhexose	C_15_H_18_O_9_	341.0877	341.0877 (27.6), 281.0659 (1.8), 251.0557 (0.9), 221.0447 (1.6), 161.0231 (100), 135.0438 (6.4)	2.60	−0.279	1,2,3,4,5,6
**7.**	caffeic acid O-dihexoside	C_21_H_28_O_14_	503.1412	503.1412 (72.8), 179.0341 (15.9), 161.0232 (100), 135.0437 (9.3), 133.0281 (31.4)	2.76	1.752	1,2,3,4,5
**8.**	caffeic acid O-dihexoside isomer	C_21_H_28_O_14_	503.1415	503.1415 (100), 341.0888 (3.5), 323.0760 (9.3), 281.0680 (16.9), 251.0563 (9.9), 221.0443 (4.1), 179.0338 (27.3), 161.0231 (80.5), 135.0437 (25.8), 133.0280 (23.3)	3.14	1.076	1,2,3,4,5,6
**9.**	chlorogenic (5-caffeoylquinic) acid ^a^	C_16_H_18_O_9_	353.0896	353.0896 (4.9), 191.0552 (100), 179.0340 (1.0)	3.19	4.998	1,2,3,4,5,6
**10.**	ferulic acid *O*-dihexoside	C_22_H_30_O_14_	517.1566	517.1566 (39.5), 193.0497 (100), 175.0390 (65.8), 149.0596 (4.7), 160.0153 (52.2), 134.0359 (29.9)	3.26	0.660	1,3,6
**11.**	coumaric acid-*O*-hexoside	C_15_H_18_O_8_	325.0930	325.0930 (13.3), 265.0714 (2.2), 235.0605 (0.4), 205.0500 (1.7), 163.0388 (12.9), 145.0282 (100), 119.0487 (4.7), 117.0331 (18.3)	3.31	0.490	1,2,3,4,5,6
**12.**	*p*-coumaric acid ^a^	C_9_H_8_O_3_	163.0387	163.0387 (80.35), 135.0437 (100), 119.0487 (63.6)	3.34	−8.510	1,2,3,4,5,6
**13.**	caffeic acid ^a^	C_9_H_8_O_4_	179.0339	179.0339 (20.5), 135.0437 (100), 117.0332 (0.64), 107.0489 (1.4)	3.55	−5.932	1,2,3,4,5,6
**14.**	ferulic acid *O*-hexoside	C_16_H_20_O_9_	355.1036	355.1036 (18.1), 295.0817 (2.1), 265.0713 (0.7), 235.0609 (3.6), 193.0497 (22.1), 175.0390 (100), 160.0154 (50.5), 149.0593 (1.1), 134.0593 (1.1), 132.0203 (12.1)	3.71	0.351	1,2,3,4,5,6
**15.**	sinapic acid *O*-dihexoside	C_23_H_32_O_15_	547.1669	547.1669 (58.7), 487.1464 (1.3), 223.0607 (100), 205.0499 (71.9), 190.0263 (62.3), 175.0026 (34.1), 149.0230 (23.4), 119.0124 (6.3)	3.80	0.158	1,3,4,5,6
**16.**	sinapic acid *O*-hexoside	C_17_H_22_O_10_	385.1143	385.1143 (29.6), 325.0927 (1.8), 295.0809 (0.7), 265.0717 (3.5), 223.0607 (22.9), 205.0499 (100), 190.0262 (40.1), 175.0026 (27.7), 149.0232 (4.2), 119.0124 (4.2)	3.82	0.701	1,2,3,4,5,6
**17.**	vanillic acid *O*-hexoside	C_14_H_18_O_9_	329.0888	329.1144 (100), 285.1242 (43.7), 167.0603 (82.7)	4.03	2.962	1,2,3,4,5,6
**18.**	*o*-coumaric acid ^a^	C_9_H_8_O_3_	163.0389	163.0389 (9.9), 135.0438 (1.3), 119.0487 (100)	4.56	−7.099	1,2,3,4,5,6
**19.**	ferulic acid ^a^	C_10_H_10_O_4_	193.0497	193.0497 (100), 178.0259 (3.5), 161.0232 (20.1), 134.0361 (7.6), 133.0281 (12.5),	6.51	−4.825	1,2,3,4,5,6
**Flavonoids**							
**20.**	luteolin 6,8-*C*-hexoside	C_27_H_30_O_16_	609.1468	609.1468 (100), 519.1131 (4.7), 489.1039 (16.4), 471.0955 (2.4), 429.0829 (7.5), 399.0723 (27.4), 369.0618 (29.4), 339.0516 (3.5), 311.0564 (4.6), 175.0392 (1.74), 133.0280 (6.01)	3.61	1.120	1,2,4,5
**21.**	luteolin *C*,*O*-dihexoside	C_27_H_30_O_16_	609.1468	609.1468 (100), 489.1036 (1.9), 447.0934 (22.9), 369.0618 (1.8), 357.0615 (13.6), 327.0511 (49.2), 313.0354 (13.3), 285.0401 (3.9), 299.0558 (10.1), 298.0408 (5.7), 175.0392 (1.5), 133.0280 (4.9)	3.85	1.120	1,2,4,5,6
**22.**	apigenin 6,8-di*C*-hexoside	C_27_H_30_O_15_	593.1517	593.1517 (100), 503.1192 (3.4), 473.1095 (15.6), 413.0893 (0.9), 395.0784 (2.4), 383.0778 (19.8), 353.0668 (30.8), 325.0719 (1.2), 297.0766 (11.9), 161.0232 (1.2), 117.0331 (3.4),	4.01	0.601	1,2,3,4,5,6
**23.**	luteolin *C*,*O*-hexoside isomer	C_27_H_30_O_16_	609.1466	609.1466 (100), 489.1053 (1.1), 447.0929 (13.4), 369.0617 (1.5), 357.0616 (23.8), 327.0511 (78.8), 313.0352 (9.3), 285.0400 (3.8), 299.0565 (11.2), 298.0487 (7.8), 175.0387 (1.8), 133.0282 (8.8)	4.07	0.824	1,2,4,5,6
**24.**	luteolin 6-*C*-hexoside-8-*C*-pentoside	C_26_H_28_O_15_	579.1362	579.1362 (100), 561.1286 (0.9), 519.1138 (2.7), 489.1041 (16.6), 429.0825 (7.9), 411.0732 (2.9), 399.0724 (29.9), 381.0612 (1.9), 369.0617 (28.8), 339.0505(4.2), 311.0563 (3.9), 299.0557 (1.7), 298.0483 (4.44), 175.0390 (1.6), 133.0281 (6.0)	4.10	1.289	1,2,3,4,5,6
**25.**	saponarin ^a^	C_27_H_30_O_15_	593.1512	593.1517 (100), 473.1094 (4.4), 431.0982 (8.4), 341.0666 (3.8), 311.0563 (28.2), 297.0403 (19.3), 269.0458 (3.5), 225.0546 (0.4), 161.0237 (1.9), 117.0332 (3.5)	4.37	0.804	1,2,3,4,5,6
**26.**	apigenin 6-*C*-hexoside-8-*C*-pentoside	C_26_H_28_O_14_	563.1406	563.1411 (100), 503.1190 (0.8), 473.1087 (11.4), 443.0981 (14.7), 425.0881 (0.8), 413.0887 (2.5), 383.0779 (12.9), 365.0659 (1.5), 353.0669 (20.7), 325.0717 (2.1), 323.0574 (1.7), 297.0769 (10.2), 283.0611 (2.6), 175.0388 (0.8), 135.0434 (1.8), 117.0331 (3.7)	4.50	0.748	1,2,3,4,5,6
**27.**	homoorientin ^a^	C_21_H_20_O_11_	447.0936	447.0936 (100), 369.0632 (2.2), 357.0618 (37.3), 339.0519 (3.7), 327.0511 (59.3), 311.0558 (3.1), 299.0561 (9.5), 298.0470 (37.3), 297.0406 (12.6), 285.0407 (7.7), 133.0282 (12.8), 175.0393 (2.5)	4.53	0.437	1,2,3,4,5,6
**28.**	chrysoeriol *C*-*O*-dihexoside	C_28_H_32_O_16_	623.1625	623.1625 (100), 503.1204 (4.5), 461.1087 (4.2), 371.0773 (2.2), 341.0665 (31.4), 327.0514 (16.2), 312.0276 (11.6), 299.0542 (1.9), 298.0408 (8.9), 340.0584 (0.5), 298.0480 (8.9), 284.0330 (5.2), 269.0457 (14.3), 163.0025 (0.5), 178.9963 (0.43)	4.60	1.255	1,2,3,4,5,6
**29.**	luteolin 7-*O*-sophoroside-6-*C*-hexoside	C_36_H_36_O_19_	771.1778	771.1786 (100), 447.0932 (34.8), 429.0760 (1.1), 369.0597 (1.1), 357.0617 (27.0), 339.0501 (4.3), 327.0510 (80.4), 299.0560 (29.3), 285.0400 (4.4), 269.0493 (0.9), 297.0408 (8.9), 133.0280 (17.6)	4.61	1.022	2
**30.**	orientin ^a^	C_21_H_20_O_11_	447.0937	447.0932 (95.5), 369.0605 (3.0), 357.0616 (32.5), 339.0504 (2.8), 327.0509 (100), 311.0558 (0.7), 299.0560 (10.0), 298.0479 (6.8), 297.0402 (12.8), 285.0399 (4.9), 165.0181 (2.1), 133.0281 (17.7), 107.0123 (0.4)	4.68	−0.256	1,2,3,4,5,6
**31.**	apigenin 6-*C*-hexoside-8-*C*-pentoside isomer	C_26_H_28_O_14_	563.1408	563.1408 (100), 503.1183 (0.4), 473.1093 (12.2), 443.0884 (19.3), 413.0893 (2.1), 383.0776 (13.7), 353.0667 (23.4), 325.0721 (2.2), 311.0569 (1.8), 297.0764 (10.4), 283.0612 (2.6), 282.0531 (1.5), 281.0451 (0.6), 175.0387 (1.0), 135.044 (1.4), 117.0331 (4.1)	4.76	0.322	2,4,5,6
**32.**	apigenin 2″-*O*-hexosyl-6-*C*-hexoside	C_27_H_30_O_15_	593.1512	593.1516 (100), 413.0876 (3.8), 395.0770 (4.6), 377.0675 (3.0), 353.0670 (21.6), 325.0708 (2.4), 311.0563 (74.3), 283.0612 (22.7), 293.0469 (3.2), 282.0536 (1.5), 281.0451 (3.8), 269.0457 (0.3), 175.0024 (0.6), 117.0330 (5.5)	4.88	0.601	1,2,3,4,5,6
**33.**	apigenin *O*-pentosyl-6-*C*-hexoside	C_26_H_28_O_14_	563.1406	563.1411 (100), 443.0986 (08), 383.0772 (2.0), 353.0669 (4.3), 341.0665 (28.1), 323.0561 (2.7), 311.0562 (89.7), 283.0611 (29.5), 282.0554 (1.2), 281.0454 (5.5), 269.0451 (2.0), 237.0553 (0.6), 121.0280 (0.4), 117.0331 (6.6)	4.99	0.748	1,2,3,5,6
**34.**	luteolin *O*-synapoylhexosyl-6-*C*-hexoside	C_38_H_40_O_20_	815.2040	815.2053 (100), 447.0936 (21.0), 357.0616 (18.5), 341.0665 (24.3), 327.0512 (79.7), 313.0356 (11.1), 299.0558 (17.1), 298.0477 (15.9), 297.0406 (10.4), 285.0399 (4.1), 133.0279 (11.1)	5.02	1.574	1,2,3,4,5,6
**35.**	rutin ^a^	C_27_H_30_O_16_	609.1467	609.1467 (100), 301.0349 (36.2), 300.0275 (67.0), 271.0247 (35.0), 255.0294 (14.4), 227.0343 (2.5), 211.0391 (1.4), 199.0388 (0.4), 178.9975 (2.4), 163.0021 (0.6), 151.0023 (5.7), 135.0069 (0.6), 107.0123 (2.2)	5.07	0.923	2,3,4,5,6
**36.**	apigenin *O*-caffeoylhexosyl-8-*C*-hexoside	C_36_H_36_O_18_	755.1829	755.1838 (92.7), 431.0985 (45.1), 413.0886 (1.6), 353.0638 (2.1), 341.0667 (27.1), 323.0538 (2.7), 311.0564 (100), 283.0612 (40.1), 281.0453 (4.5), 269.0436 (1.4), 161.0231 (15.2), 133.0278 (8.9), 117.0391 (7.8)	5.13	1.262	1,2,4,5
**37.**	isovitexin ^a^	C_21_H_20_O_10_	431.0985	431.0985 (100), 412.0894 (1.4), 341.0669 (18.7), 323.0570 (4.1), 311.0565 (58.4), 283.0614 (18.2), 269.0452 (2.9), 117.0330 (7.7), 161.0233 (2.4), 135.0441 (1.7)	5.14	0.394	1,2,3,4,5,6
**38.**	luteolin *O*-feruloylhexosyl-8-*C*-hexoside	C_37_H_38_O_19_	785.1935	785.1940 (100), 447.0940 (28.4), 369.0600 (2.1), 357.0618 (24.0), 339.0502 (1.9), 327.0510 (92.8), 299.0566 (23.2), 298.0485 (14.1), 297.0408 (10.1), 341.0658 (18.4), 285.0413 (2.6), 175.0392 (5.0), 133.0282 (13.0)	5.20	0.736	1,2,4,5
**39.**	luteolin 7-*O*-coumaroylhexosyl-6-*C*-hexoside	C_36_H_36_O_18_	755.1829	755.1837 (100), 447.0936 (30.7), 369.0602 (2.2), 357.0618 (23.3), 339.0506 (2.7), 327.0511 (76.6), 299.0562 (18.4), 298.0480 (6.6), 297.0401 (5.2), 285.0393 (2.9), 163.0387 (2.2), 145.0278 (3.2), 133.0279 (8.7), 119.0488 (0.6), 117.0331 (2.5),	5.27	1.103	1,2,4,5
**40.**	eryodictiol 7-*O*-hexoside	C_21_H_22_O_11_	449.1089	449.1094 (14.8), 287.0561 (100), 151.0023 (61.8), 135.0437 (50.5), 125.0228 (4.1), 107.0123 (13.0)	5.27	1.058	1,2,3,4,5,6
**41.**	chrysoeriol 7-*O*-caffeoylhexosyl-8-*C*-hexoside	C_37_H_38_O_19_	785.1935	785.1936 (91.8), 461.1106 (36.0), 371.0775 (29.5), 341.0666 (100), 327.0528 (12.7), 298.0479 (60.7), 299.0531 (6.9), 297.0406 (5.5), 161.0232 (13.3), 133.0283 (11.3)	5.28	0.189	1,2,4,5
**42.**	isoquercitrin ^a^	C_21_H_20_O_12_	463.0882	463.0885 (100), 301.0349 (44.1), 300.0276 (75.1), 271.0248 (37.1), 255.0296 (0.7), 243.0299 (9.3), 227.0342 (2.4), 211.0391 (0.6), 199.0392 (0.5), 178.9969 (3.0), 163.0027 (1.9), 151.0024 (5.3), 135.0072 (0.5), 121.0280 (1.6), 107.0123 (2.8)	5.28	0.714	1,2,3,4,5,6
**43.**	luteolin-7-*O*-glucoside ^a^	C_21_H_20_O_11_	447.0937	447.0937 (100), 285.0404 (86.7), 284.0327 (36.3), 255.0302 (1.2), 227.0346 (1.9), 211.0397 (1.1), 151.0024 (4.7), 133.0282 (4.2), 107.0124 (3.3)	5.37	0.840	1,2,3,4,5,6
**44.**	chrysoeriol 6-*C*-hexoside	C_22_H_22_O_11_	461.1089	461.1093 (100), 371.0422 (21.2), 353.0674 (2.8), 341.0665 (69.0), 313.0727 (3.2), 312.0609 (0.5), 299.0520 (4.8), 298.0481 (14.1), 297.0404 (13.3), 284.0327 (1.7), 269.0455 (2.9), 255.0294 (1.4), 133.0284 (2.2)	5.43	0.814	1,2,3,4,5,6
**45.**	apigenin 7-*O*-synapoylhexosyl-8-*C*-hexoside	C_38_H_40_O_19_	799.2103	799.2103 (88.3), 431.0984 (53.7), 341.0665 (29.8), 323.0559 (3.8), 311.0563 (100), 283.0610 (44.5), 282.0533 (9.9), 281.0463 (7.4), 190.0265 (4.8), 175.0025 (1.8)	5.46	1.537	1,2,4,5
**46.**	chrysoeriol 7-*O*-dihexoside	C_28_H_32_O_16_	623.1620	623.1620 (27.1), 299.0561 (100), 284.0326 (40.6), 255.0289 (4.1), 151.0026 (0.9)	5.49	0.469	4, 5
**47.**	kaempferol 3-*O*-rutinoside ^a^	C_27_H_30_O_15_	593.1512	593.1518 (100), 285.0403 (82.5), 284.0327 (47.6), 255.0298 (37.7), 227.0345 (24.2), 211.0397 (2.5), 135.0071 (1.5), 151.0024 (1.8), 107.0124 (2.4)	5.63	1.006	1,2,4,5,6
**48.**	apigenin *O*-feruloylhexosyl-8-*C*-hexoside	C_38_H_38_O_18_	769.200	769.1996 (83.5), 431.0985 (52.3), 353.0680 (3.5), 341.0666 (23.9), 323.0564 (5.2), 311.0563 (100), 283.0611 (41.8), 282.0521 (4.6), 281.0452 (6.7), 269.0443 (2.0), 175.0391 (2.1), 117.0332 (9.1)	5.70	1.382	1,2,3,4,5,6
**49.**	chrysoeriol *O*-feruloylhexoside-8-*C*-hexoside	C_38_H_40_O_19_	799.2091	799.2111 (60.9), 461.1094 (83.2), 371.0762 (31.7), 353.0687 (2.1), 341.0667 (100), 309.0394 (7.2), 299.0500 (2.9), 298.0483 (54.9), 297.0399 (13.5), 175.0387 (2.8), 160.0146 (1.7), 132.0201 (2.4)	5.78	2.525	1,2,4,5
**50.**	isorhamnetin 3-*O*-rutinoside ^a^	C_28_H_32_O_18_	623.1618	623.1622 (100), 315.0509 (95.2), 300.0269 (13.6), 299.0201 (15.1), 271.0248 (33.7), 255.0295 (12.3), 243.0297 (15.8), 227.0345 (4.3), 1561.0022 (1.6)	5.78	0.758	1,3,4,5,6
**51.**	apigenin *O*-coumaroylhexosyl-8-*C*-hexoside	C_36_H_36_O_17_	739.1880	739.1893 (87.0), 431.0983 (39.6), 413.0881 (1.1), 253.0668 (2.6), 341.0667 (25.1), 323.0562 (5.9), 311.0564 (100), 283.0612 (44.0), 282.0534 (2.4), 281.0468 (4.4), 145.0282 (5.5), 117.0331 (10.9)	5.80	1.836	1, 2, 3, 4, 6
**52.**	kaempferol 3-*O*-glucoside ^a^	C_21_H_20_O_11_	447.0934	447.0935(100), 285.0398 (21.9), 284.0327 (52.1), 255.0297 (39.5), 227.0346 (39.1), 211.0394 (0.7), 178.9966 (0.7), 151.0027 (1.8), 107.0126 (0.6)	5.85	0.482	1,2,3,4,5,6
**53.**	chrysoeriol *O*-coumaroylhexoside-8-*C*-hexoside	C_37_H_38_O_18_	769.2000	769.2000 (83.7), 461.1091 (62.3), 371.0771 (30.1), 341.0667 (100), 353.0651 (1.5), 309.0401 (6.6), 298.0481 (67.2), 299.0519 (6.7), 297.0400 (14.6), 284.0330 (2.1),	5.92	1.837	1,2,4,5
**54.**	apigenin 7-*O*-neohesperidoside	C_27_H_30_O_14_	577.1568	577.1568 (69.4), 431.0989 (0.5), 413.0882 (1.0), 269.0454 (100), 211.0390 (0.8), 151.0023 (2.2), 149.0230 (0.8), 117.0330 (3.5), 107.0121 (1.8)	6.00	0.903	1,2,3,4,5,6
**55.**	apigenin 7-*O*-glucoside ^a^	C_21_H_20_O_10_	431.0985	431.0985 (100), 269.0449 (26.9), 268.0378 (61.1), 211.0394 (1.8), 151.0025 (2.9), 149.0228 (1.2), 107.0124 (2.5), 117.0331 (2.1)	6.04	0.325	1,2,3,4,5,6
**56.**	chrysoeriol 7-*O*-rutinoside	C_28_H_32_O_15_	607.1668	607.1676 (89.6), 299.0561 (100), 284.0327 (43.3), 255.0299 (43.3)	6.20	1.246	1,2,3,4,5,6
**57.**	chrysoeriol 7-*O*-hexoside	C_22_H_22_O_11_	461.1081	461.1081 (100), 446.0857 (24.6), 371.0756 (0.2), 341.0648 (0.6), 300.0590 (0.9), 299.0555 (9.3), 298.0482 (12.6), 283.0248 (18.1), 255.0297 (68.6), 227.0348 (0.3), 211.0398 (0.2), 151.0027 (0.7), 117.0331 (1.7), 107.0124 (0.1)	6.25	−1.832	1,2,3,4,5,6
**58.**	cirsiliol *O*-hexoside	C_23_H_24_O_12_	491.1195	491.1191 (100), 476.0944 (21.6), 461.0748 (5.1), 329.0676 (3.7), 328.0594 (6.6), 314.0442 (3.8), 313.0351 (6.4), 299.0198 (6.1), 285.0397 (12.4), 243.0292 (10.3)	6.30	−0.854	1,2,4,5,6
**59.**	luteolin 2″-*O*-coumaroyl-6-*C*-hexoside	C_30_H_26_O_13_	593.1301	593.1309 (100), 473.0900 (2.3), 447.0922 (31.5), 429.0841 (13.5), 357.0603 (13.6), 339.0526 (2.1), 327.0511 (38.8), 309.0406 (46.4), 299.0560 (12.9), 297.0399 (2.0), 285.0432 (1.4)	6.71	1.342	1,2,3,6
**60.**	luteolin *O*-coumaroyldeoxyhexosyl-6-hexoside	C_36_H_36_O_17_	739.1880	739.1893 (100), 447.0944 (12.3), 369.0611 (1.2), 357.0617 (13.8), 339.0518 (1.7), 327.0509 (58.8), 313.0355 (20.1), 299.0552 (13.8), 298.0485 (10.1), 297.0402 (7.0), 285.0396 (5.9), 269.0457 (1.1), 163.0396 (1.3), 151.0024 (0.8), 133.0281 (8.9)	6.75	1.836	1,2,3,4,5
**61.**	apigenin 2″-*O*-coumaroyl-6-*C*-hexoside	C_30_H_26_O_12_	577.1351	577.1365 (7.4), 431.0986 (100), 413.0882 (77.5), 341.0669 (21.3), 323.0593 (3.0), 311.0505 (65.4), 293.0457 (96.2), 283.1061 (35.7), 269.0435 (1.6), 175.0026 (15.2), 163.0389 (20.3), 119.0487 (34.1), 117.0332 (12.7)	7.42	2.410	1,2,5,6
**62.**	apigenin *O*-coumaroyldeoxyhexosyl-6-*C*-hexoside	C_36_H_36_O_16_	723.1931	723.1941 (100), 431.0979 (8.5), 341.0677 (5.4), 311.0564 (53.5), 297.0403 (27.2), 283.0611 (20.3), 282.0534 (9.6), 281.0454 (6.0), 269.0457 (4.9), 163.0387 (2.1), 135.0436 (1.0), 117.0330 (8.1)	7.47	1.427	2,4,5
**63.**	apigenin ^a^	C_15_H_10_O_5_	269.0455	269.0455 (100), 227.0363 (1.3), 151.0027 (6.5), 149.0229 (4.4), 117.0331 (18.6), 107.0123 (4.5),	8.62	−0.917	1,2,3,4,5,6
**64.**	luteolin ^a^	C_15_H_10_O_6_	285.0404	285.0404 (100), 241.0493 (0.4), 151.0025 (4.1), 149.0233 (1.9), 133.0280 (20.4), 107.0124 (3.6)	8.91	−0.071	1,2,3,4,5,6
**65.**	chrysoeriol	C_16_H_12_O_6_	299.0561	299.0561 (100), 284.0327 (81.0), 256.0375 (16.6), 227.0346 (2.9), 151.0025 (3.1), 107.0123 (2.2)	8.91	−0.071	1,2,3,4,5,6
**Amino acids and derivatives**							
**66.**	N-hexosylglutamic acid	C_11_H_17_O_8_N	290.0883	290.0883 (8.82), 272.0277 (2.2), 254.0680 (1.3), 230.0668 (2.9), 200.0556 (44.1), 170.0448 (8.8), 128.0338 (100), 84.0438 (1.7)	0.72	2.615	1,2,3,4,5,6
**67.**	N-hexosylvaline	C_11_H_21_O_7_N	278.1246	278.1246 (1.1), 260.1139 (1.1), 188.0918 (6.2), 158.0810 (4.3), 146.0442 (0.5), 116.0701 (100), 101.0228 (8.8), 85.0280 (0.3)	0.90	0.161	1,3,4,5,6
**68.**	N-hexosyltyrosine	C_15_H_21_O_8_N	342.1197	342.1171 (3.8), 324.1084 (0.6), 282.0967 (0.5), 252.0877 (11.5), 222.0766 (9.1), 180.0656 (100), 163.0388 (11.1), 119.0487 (10.2)	0.92	0.644	1,2,3,4,5,6
**69.**	N-hexosylleucine	C_15_H_23_O_7_N	292.1400	292.1400 (1.3), 274.1300 (0.4), 232.1179 (0.3), 202.1078 (7.9), 172.0968 (3.1), 152.1058 (0.3), 130.0859 (100)	1.07	1.106	1,2,3,4,5,6
**70.**	N-hexosylphenylalanine	C_15_H_21_O_7_N	326.1248	326.1248 (1.8), 308.1145 (1.1), 266.1046 (0.6), 236.0925 (11.2), 206.0816 (6.1), 186.0916 (0.4), 164.0705 (100), 147.0438 (18.9), 119.0485 (1.8), 101.0229 (14.2)	1.53	0.720	1,2,3,4,5,6
**71.**	phenylalanine	C_9_H_11_O_2_N	164.0705	164.0705 (53.3), 147.0438 (100), 146.0594 (0.5), 135.0437 (13.0), 119.0489 (8.9), 103.9187 (2.2)	1.54	−7.508	1,2,3,4,5,6
**72.**	N-hexosyltryptophan	C_17_H_22_O_7_N_2_	365.1360	365.1357 (0.6), 275.1040 (10.5), 245.0924 (3.3), 203.0801 (100), 142.0647 (5.9), 159.0915 (2.3)	2.36	1.714	1,2,3,4,5,6
**73.**	tryptophan	C_11_H_12_O_2_N_2_	203.0818	203.0818 (71.0), 186.0548 (4.4), 159.0916 (19.9), 142.0649 (25.7), 116.0491 (100), 74.0231 (31.2), 72.0075 (30.9)	2.48	2.766	1,2,3,4,5,6
**74.**	γ-glutamyl-leucine	C_11_H_20_O_5_N_2_	259.1300	241.1191 (20.0), 223.1083 (10.5), 215.1400 (0.4), 197.1287 (11.1), 130.0859 (64.9), 128.0339 (100), 127.0499 (0.8)	2.82	0.212	1,2,3,4,5,6
**Fatty acids**							
**75.**	trihydroxyoctadecadienoic acid	C_18_H_32_O_5_	327.2177	327.2177 (100), 309.2070 (0.8), 291.1971 (3.90), 247.2048 (0.5), 239.1286 (3.1), 229.1440 (3.9), 211.1331 (20.4), 183.1382 (2.2), 171.1014 (6.4), 155.1071 (0.6), 127.1112 (0.7), 97.0643 (3.3), 85.0277 (3.9), 70.1990 (0.9)	9.13	0.130	1,2,3,4,5,6
**76.**	trihydroxyotadecenoic acid	C_18_H_34_O_5_	329.2334	329.2334 (100), 311.2224 (1.0), 293.2121 (0.9), 229.1439 (18.8), 211.1332 (24.6), 199.1344 (0.3), 183.1379 (2.9), 171.1014 (4.1), 155.1066 (0.3), 139.111 (1.42), 127.1111 (1.1), 99.0799 (3.6), 83.0488 (0.3), 70.1988 (0.3)	9.78	0.069	1,2,3,4,5,6
**77.**	trihydroxyoctadecatrienoic acid	C_18_H_30_O_5_	325.2019	325.2019 (100), 307.1915 (19.7), 289.1807 (1.9), 263.2015 (12.5), 245.1904 (0.4), 235.2053 (0.9), 209.0526 (0.2), 169.1218 (0.4), 137.0956 (3.9), 125.0957 (2.9), 97.0642 (0.3), 83.0484 (0.9), 57.0328 (1.3)	11.85	−0.453	1,2,3,4,5,6
**78.**	dihydroxyoctadecadienoic acid	C_18_H_32_O_4_	311.2229	311.2229 (100), 293.2127 (6.6), 275.2008 (4.0), 235.1698 (4.16), 223.1697 (39.9), 208.2670 (0.3), 196.1057 (1.5), 113.0954 (1.1), 87.0435 (12.4), 57.0330 (5.3)	12.51	0.377	1,2,3,4,5,6
**79.**	dihydroxyoctadecenoic acid	C_18_H_34_O_4_	313.2385	313.2385 (100), 295.2277 (9.5), 277.2174 (2.6), 209.1976 (0.2), 195.1382 (2.7), 183.1380 (25.5), 129.0907 (16.5), 99.0799 (10.5)	13.46	0.183	1,2,3,4,5,6
**80.**	dihydroxyoctadecenoic acid	C_18_H_34_O_4_	313.2385	313.2385 (100), 295.2277 (5.5), 277.2174 (4.6), 201.1124 (43.2), 171.1014 (5.9), 155.1065 (1.0), 139.1116 (0.7), 127.1113 (3.9)	13.72	0.183	1,2,3,4,5,6
**81.**	dihydroxyoctadecatrienoic acid	C_18_H_30_O_4_	309.2071	309.2071 (100), 291.1966 (28.6), 273.1864 (5.00), 263.2047 (0.4), 247.2063 (26.6), 139.1116 (0.6), 70.2070 (0.8), 57.0329 (1.9)	14.61	−0.138	1,2,3,4,5,6
**82.**	dihydroxyoctadecanoic acid	C_18_H_36_O_4_	315.2540	315.2540 (100), 297.2435 (5.6), 201.1130 (0.3), 171.1015 (0.9), 155.1064 (0.2), 141.1269 (2.3), 127.1113 (1.1)	14.85	−0.385	1,2,3,4,5,6
**83.**	linoleic acid	C_18_H_32_O_2_	279.2327	279.2327 (100), 136.1251 (0.4), 70.0576 (0.7)	21.96	−1.05	3,6

^a^—compare to reference standard; 1—*E. intortum* leaves; 2—*E. intortum* flowers; 3—*E. intortum* tuber; 4—*E. spiculatum* leaves; 5—*E. spiculatum* flowers; 6—*E. spiculatum* tuber.

**Table 3:** Antioxidant properties of the tested extracts.

Species	Parts	DPPH (mg TE/g)	ABTS (mg TE/g)	CUPRAC (mg TE/g)	FRAP (mg TE/g)	PBD (mmol TE/g)	MCA (mg EDTAE/g)
*Eminium intortum*	Leaves	32.20 ± 1.26 ^a^	52.10 ± 0.83 ^a^	88.27 ± 1.49 ^a^	33.13 ± 0.68 ^a^	0.96 ± 0.01 ^b^	63.43 ± 0.70 ^a^
	Flowers	27.87 ± 0.75 ^b^	40.44 ± 1.20 ^c^	55.29 ± 1.78 ^c^	24.67 ± 1.22 ^b^	1.38 ± 0.08 ^a^	36.05 ± 1.22 ^b^
	Tubers	26.90 ± 1.61 ^b^	46.80 ± 0.86 ^b^	71.51 ± 1.93 ^b^	32.58 ± 4.02 ^a^	1.48 ± 0.15 ^a^	39.15 ± 2.79 ^b^
*Eminium spiculatum*	Leaves	26.58 ± 1.00 ^b^	54.34 ± 0.53 ^a^	86.27 ± 2.74 ^a^	29.90 ± 0.75 ^b^	1.08 ± 0.03 ^b^	61.55 ± 3.97 ^a^
	Flowers	20.73 ± 1.51 ^c^	48.45 ± 1.96 ^b^	70.06 ± 3.03 ^b^	30.92 ± 0.21 ^b^	1.23 ± 0.05 ^a^	30.92 ± 0.48 ^c^
	Tubers	33.67 ± 1.26 ^a^	51.86 ± 2.14 ^ab^	75.43 ± 4.47 ^b^	41.33 ± 1.25 ^a^	1.02 ± 0.07 ^b^	53.00 ± 1.88 ^b^

Values are reported as mean ± SD of three parallel measurements. TE: Trolox equivalent; PBD: Phosphomolybdenum assay; MCA: Metal chelating assay; EDTAE: EDTA equivalent. Different letters indicate significant differences between the tested extracts (*p* < 0.05).

**Table 4:** Enzyme inhibitory effect of the tested extracts.

Species	Parts	AChE (mg GALAE/g)	BChE (mg GALAE/g)	Tyrosinase (mg KAE/g)	Amylase (mmol ACAE/g)	Glucosidase (mmol ACAE/g)
*Eminium intortum*	Leaves	1.27 ± 0.09 ^c^	na	35.37 ± 0.74 ^c^	0.30 ± 0.01 ^b^	0.35 ± 0.02 ^c^
	Flowers	2.25 ± 0.07 ^b^	0.67 ± 0.08 ^b^	40.66 ± 0.61 ^b^	0.31 ± 0.01 ^a^	na
	Tubers	2.64 ± 0.01 ^a^	2.01 ± 0.07 ^a^	48.13 ± 0.24 ^a^	0.26 ± 0.01 ^c^	na
*Eminium spiculatum*	Leaves	2.64 ± 0.10 ^b^	1.11 ± 0.20 ^b^	42.58 ± 1.28 ^b^	0.27 ± 0.01 ^a^	0.99 ± 0.02 ^a^
	Flowers	2.72 ± 0.03 ^a^	1.89 ± 0.07 ^a^	37.74 ± 1.94 ^c^	0.24 ± 0.01 ^b^	0.89 ± 0.01 ^b^
	Tubers	2.66 ± 0.05 ^a^	1.69 ± 0.13 ^a^	50.73 ± 2.29 ^a^	0.24 ± 0.01 ^b^	na

Values are reported as mean ± SD of three parallel measurements. GALAE: Galantamine equivalent; KAE: Kojic acid equivalent; ACAE: Acarbose equivalent; na: not active. Different letters indicate significant differences between the tested extracts (*p* < 0.05).

## Data Availability

Not applicable.
